# Regional Reproducibility of BOLD Calibration Parameter *M*, OEF and Resting-State CMRO_2_ Measurements with QUO2 MRI

**DOI:** 10.1371/journal.pone.0163071

**Published:** 2016-09-20

**Authors:** Isabelle Lajoie, Felipe B. Tancredi, Richard D. Hoge

**Affiliations:** 1 Département de physiologie moléculaire et intégrative, Institut de génie biomédical, Université de Montréal, Montreal, Quebec, Canada; 2 Department of Neurology and Neurosurgery, Montreal Neurological Institute, McGill University, Montreal, Quebec, Canada; 3 Departamento de Radiologia, Centro de Pesquisa em Imagem, Hospital Israelita Albert Einstein, São Palo, SP, Brazil; Universitair Medisch Centrum Utrecht, NETHERLANDS

## Abstract

The current generation of calibrated MRI methods goes beyond simple localization of task-related responses to allow the mapping of resting-state cerebral metabolic rate of oxygen (CMRO_2_) in micromolar units and estimation of oxygen extraction fraction (OEF). Prior to the adoption of such techniques in neuroscience research applications, knowledge about the precision and accuracy of absolute estimates of CMRO_2_ and OEF is crucial and remains unexplored to this day. In this study, we addressed the question of methodological precision by assessing the regional inter-subject variance and intra-subject reproducibility of the BOLD calibration parameter *M*, OEF, O_2_ delivery and absolute CMRO_2_ estimates derived from a state-of-the-art calibrated BOLD technique, the QUantitative O2 (QUO2) approach. We acquired simultaneous measurements of CBF and R2* at rest and during periods of hypercapnia (HC) and hyperoxia (HO) on two separate scan sessions within 24 hours using a clinical 3 T MRI scanner. Maps of *M*, OEF, oxygen delivery and CMRO_2_, were estimated from the measured end-tidal O_2_, CBF_0,_ CBF_HC/HO_ and R2*_HC/HO_. Variability was assessed by computing the between-subject coefficients of variation (bwCV) and within-subject CV (wsCV) in seven ROIs. All tests GM-averaged values of CBF_0_, *M*, OEF, O_2_ delivery and CMRO_2_ were: 49.5 ± 6.4 mL/100 g/min, 4.69 ± 0.91%, 0.37 ± 0.06, 377 ± 51 μmol/100 g/min and 143 ± 34 μmol/100 g/min respectively. The variability of parameter estimates was found to be the lowest when averaged throughout all GM, with general trends toward higher CVs when averaged over smaller regions. Among the MRI measurements, the most reproducible across scans was R2*_0_ (wsCV_GM_ = 0.33%) along with CBF_0_ (wsCV_GM_ = 3.88%) and R2*_HC_ (wsCV_GM_ = 6.7%). CBF_HC_ and R2*_HO_ were found to have a higher intra-subject variability (wsCV_GM_ = 22.4% and wsCV_GM_ = 16% respectively), which is likely due to propagation of random measurement errors, especially for CBF_HC_ due to the low contrast-to-noise ratio intrinsic to ASL. Reproducibility of the QUO2 derived estimates were computed, yielding a GM intra-subject reproducibility of 3.87% for O_2_ delivery, 16.8% for the *M* value, 13.6% for OEF and 15.2% for CMRO_2_. Although these results focus on the precision of the QUO2 method, rather than the accuracy, the information will be useful for calculation of statistical power in future validation studies and ultimately for research applications of the method. The higher test-retest variability for the more extensively modeled parameters (*M*, OEF, and CMRO_2_) highlights the need for further improvement of acquisition methods to reduce noise levels.

## Introduction

Mapping of resting metabolism in the brain is of considerable interest for diagnostic and research applications. Until recently, positron emission tomography (PET) using a triple injection of radio-labeled O_2_ was the only imaging method for measuring cerebral metabolic rate of O_2_ consumption (CMRO_2_) [1]. The PET method requires exposure to ionizing radiation, arterial sampling, and access to an on-site cyclotron to produce the short-lived ^15^O-labeled tracers, limitations that have led to the development of magnetic resonance imaging (MRI) techniques to measure O_2_ consumption [2–4].

The approach proposed by our team, Quantitative O_2_ (QUO2) MRI is based on respiratory calibration of the BOLD signal, in which the oxygen extraction fraction (OEF) at rest is determined using hypercapnia (HC) and hyperoxia (HO). During the respiratory manipulation, we monitor end-tidal O2 (ETO_2_) levels and use dual-echo ASL to measure BOLD and cerebral blood flow (CBF) simultaneously. ETO_2_, BOLD and CBF then serve as inputs to the generalized calibration model (GCM) described in Gauthier and Hoge [5], which yields a system of two equations with solutions for the BOLD calibration parameter *M*, i.e. the maximum BOLD signal increase when venous O_2_ saturation approaches 100%, and resting OEF. Multiplication of OEF by baseline CBF and arterial O_2_ content (estimated from ETO_2_ monitoring and, optionally, blood testing) gives resting CMRO_2_ in absolute units, e.g. μmol/100 g/min.

An initial proof-of-concept of the QUO2 method was presented in Gauthier and Hoge [6] to obtain individual and group maps of BOLD calibration parameter *M*, resting OEF and CMRO_2_. While valid regional and group-averaged estimates of the latter parameters were obtained, individual OEF maps showed signs of instability characterized by large fluctuations in the modeled values and a lack of solution in certain regions. The stability of individual solution maps generated using this method depends on accurate and robust measures of end-tidal O_2_ and maps of fractional changes in BOLD and CBF during the respiratory manipulation. In an attempt to improve the stability and avoid circumstances where the QUO2 model cannot be solved, we have adapted the imaging and respiratory protocols used in several ways. Instead of performing two separate respiratory scans for hyperoxia and hypercapnia, we have adopted the 18-minute respiratory sequence that alternates between periods of hypercapnia and hyperoxia [2] and during which the total time dedicated to each gas manipulation is increased compared to the original protocol, improving statistical power. We developed a simple breathing circuit allowing a better control over fractional concentration of inspired gas and thus yielding improved stability of end-tidal values compared with the simple oxygen masks used in our earlier studies [7]. Combining a dual-echo Pseudo-Continuous ASL sequence (de-pCASL), the respiratory protocol and breathing circuit mentioned above, we assessed the test-retest reliability in the respiratory responses and in CBF and BOLD responses within GM [8]. In the present study, we attempted to further improve the voxel-wise image quality in single-subject maps by performing an integrated analysis on the dual-echo pCASL data. The novel analysis strategy (further described in Materials and Methods) involved 1) motion correcting the interleaved echo series using the same transformation for the two echo times; 2) applying a more holistic general linear model on the motion-corrected series to extract baseline parameters and gas responses using one regressor per combination of echo and tag/control; 3) employing both echoes information to estimate R2* rather than approximating it from the second echo; and 4) employing a more sophisticated approach to mitigate solution instabilities from isolated non-parenchymal voxels using 3D median filtering.

The aim of this study was to assess, in a small cohort of healthy individuals, the variability of the optimized QUO2 measures across and within subject in different brain regions. These results, based on the precision of the method, will help guide future developments and research application of the method. The following estimates were obtained: BOLD calibration parameter *M*, OEF, O_2_ delivery and resting-state CMRO_2_. The impact of systematic and random errors on the accuracy and precision of such estimates was also evaluated. Furthermore, these estimates were compared with CBF and BOLD-based reproducibility estimates derived from the same group of subjects and same enhanced analysis.

## Materials and Methods

Eight healthy adults were enrolled in this study (4 females, mean age: 30.5 ± 5.7 years). All participants gave written informed consent and the project was approved by the Comité mixte d’éthique de la recherche du Regroupement Neuroimagerie/Québec. They were scanned twice (referred to as Test A and Test B), within 24 hours, using the same imaging procedures and respiratory manipulation. To minimize effects of diurnal fluctuation in blood flow, all sessions was acquired between 2 PM and 6 PM [9]. The participants were asked to abstain from caffeine 3 hours prior to scanning.

### Respiratory Manipulation

For the respiratory manipulation, we adopted the gas sequence described by Bulte et al [2] with a total duration of 18 minutes. This involves two 2-min periods of hypercapnia (HC) and two 3-min periods of hyperoxia (HO). HC was followed by a 1-min normocapnic period and then the 3-min hyperoxic stimulus. HO was followed by a 3-min period of normoxia.

HC and HO blocks were induced by supplying participants with gas mixtures enriched with CO_2_ or O_2_. Participants breathed the gas mixtures through the breathing circuit developed in-house [7]. An automated system, also developed in-house, was used to deliver the gas mixtures with a reproducible time course in all scanning sessions. The system comprises 4 flow controllers (SideTrak® 840, Sierra instruments, L. Monterey, CA, USA), a ~25 mL mixing chamber, a digital interface (Sierra, FloBox^TM^ 954) to send commands to the flow controllers and a laptop computer to automate the gas mixture processes and collect behavioral data. The system’s output is connected to the breathing circuit via 10 meters of plastic tubing (BIOPAC, AFT31-MRI). Three gases were input to the flow controllers: medical air, oxygen and a 5% CO_2_ and air mixture. During the hyperoxia periods, subjects breathed a mixture of 50% pure oxygen balanced with air, yielding a fix inspired O_2_ concentration of 60% O_2_. Otherwise, participants were given medical air to breathe. Gas mixtures were administered at a rate of 20 L/min, except during transitions in inspired concentrations, during which the flow rate was increased to 50 L/min for 5.4 seconds in order to accelerate transitions.

Respiratory gases were continuously monitored using the CO2100C and O2100C modules of a BIOPAC MP150 system (BIOPAC Systems Inc., CA, USA). Gases were sampled via a 10m segment of rigid tubing (AFT31-XL, from BIOPAC System Inc.) in series with a bacterial filter (#2200/01, GVS filter technology, LA, UK) and 1’ segment of oxygen tubing attached to the sampling port of the respiratory circuit.

### Image Acquisition

Images were acquired on a clinical 3 T scanner (Siemens TIM TRIO, Siemens Medical Solutions, Erlangen, Germany) using the vendor’s 32-channel receive-only head coil. The scan session included a 5-minute anatomical acquisition (1 mm^3^ MPRAGE with TR/TE/flip angle = 2.3 seconds/3 msec/9°, 256x240 matrix, GRAPPA factor = 2), and an 18-minute functional scan using dual-echo pseudo-continuous ASL sequence (de-pCASL) [10] in order to acquire simultaneous measures of BOLD and CBF. The de-pCASL parameters were: TR/TE1/TE2/alpha = 4.12 seconds/8.4 msec/30 msec/90°, labeling duration = 2 seconds using Hanning window-shaped RF pulse with duration/space = 500 μsec/360 μsec, flip angle = 25°, slice-selective gradient = 6 mT/m, label offset = 100 mm below the center of image slab, nominal and average post-labeling delay (PLD) = 0.9 and 1.44 seconds respectively. The readout consisted of a GRE-EPI with GRAPPA factor = 2, partial sampling of k-space = 7/8, in-plane resolution of 4.5 x 4.5 mm^2^, 21 slices with 4.5 mm thickness and 0.45 mm gap.

### Respiratory Data Analysis

Analysis of the respiratory data was carried out using an in-house program developed in Matlab (MathWorks, Natick, MA, USA). An automatic extraction of the end-tidal (ET) and fixed inspired (FI) points from the continuous O_2_ and CO_2_ traces was performed. It was observed that the filter placed in series with the sampling line added an extra resistance causing an effect of low-pass filtering to the respiratory waveform. This resulted in an offset of both the ET and FI monitored pressures, i.e. an attenuation of the peak-to-peak amplitude of the waveform, which was dependent on the participant’s breathing pace. Each ET point was corrected using the average of the differences between the observed and expected FI points surrounding it. ET values were also corrected to account for an expired partial pressure of water of 47 mmHg [11]. The resting ET and changes in ET during HC and HO periods were determined by applying the linear model previously described [12]. The model is composed of a third-degree polynomial term and four regressors to represent responses to the hypercapnic and hyperoxic blocks. The offset term served to estimate the baseline ET whereas the effect size of each response regressor yielded an estimate of the associated ET change. Final ETO_2_ change to periods of HO was obtained by averaging the two ETO_2_ changes to HO. The same method was employed to compute the final ETO_2_ change to periods of HC, and ETCO_2_ responses to both gases.

The average values of ETO_2_ at baseline and during both respiratory stimuli were used to compute arterial O_2_ content (ml O_2_/ml blood) and change in the venous deoxygenated fraction ([dHb]/ [dHb]_0_) as in Chiarelli et al [13] and Gauthier et al [3]. The latter quantities are integrated to obtain the BOLD calibrated value *M*, resting OEF and CMRO_2_ as specified below.

### Imaging Data Analysis

#### Preprocessing

Analysis of functional scans was performed using in-house software implemented in C. The interleaved echo series was motion corrected with consecutive first and second echo frames sharing the same transformation matrix. The resultant series was spatially filtered (8mm FWHM 3D Gaussian kernel), had extra-cerebral voxels removed and was intensity normalized (brain mean 100). The fMRI data were then fit to a GLM to extract the label and control series of both echoes during baseline, hypercapnia and hyperoxia periods. The model used four regressors per conditions to account for both echoes label and control points, and a third-degree polynomial with an offset term representing signal drifts. We used a single-gamma HRF function with 20 seconds time-to-peak and 40 seconds width, which yielded near-exponential transitions to account for the slow response of the arterial partial pressures to the inspired gas [14]. ASL (S0) and BOLD (R2* or 1/T2*) control and label series at baseline and during gas manipulations were computed using both echoes information. ASL flow series were computed from subtraction of S0 control to S0 label series, whereas BOLD series were isolated averaging the control and label R2* series. A 3D median filtering (radius of 1 voxel) was applied on the resultant maps to minimize the impact of non-parenchymal voxels such as those containing large blood vessels.

The functional maps produced by the above analysis were then used to further reduce the impact of voxels not meeting the assumptions of the QUO2 model: Baseline T2* maps served to exclude voxels in regions degraded by susceptibility artifact (lower threshold of 30ms). Voxels in which ΔR2*_HO_ was positive were assumed to be dominated by susceptibility artifacts from adjacent nasal cavity due to the paramagnetic effect of molecular O_2_. Additional voxels with positive ΔR2*_HC_ were considered as non-parenchymal and were also excluded from the analysis. The ASL signal was converted into physiological units of flow (mL/100 g/min) as in Wang et al [15] using the constants recommended by the ISMRM Working Committee [16] and an adjusted PLD to account for slice acquisition time (PLD range for 21 slices of 900-1960ms).

During hyperoxic manipulation, the T1 of blood is altered due to an increase in plasma concentration of paramagnetic O_2_ [17]. To account for this change in blood T1, which would bias our Δ%CBF_HO_ estimation, we applied a corrective factor using the approach described in Chalela et al [18] and Zaharchuk et al [19]. The T1 of blood during hyperoxic intervals was estimated individually using the R1 and PaO_2_ relationship in rats’ blood reported in Pilkinton et al [17].

#### Computation of metabolism

For each gas challenge, the changes in the venous deoxygenated fraction, along with the change in BOLD (ΔR2*) and CBF were used as inputs to the generalized calibration model (GCM) described in Gauthier et al [5]. This yields a system of two equations with two unknowns: the BOLD calibration parameter *M* (extrapolated maximum BOLD fractional signal increase when venous O_2_ saturation approaches 100%) and OEF (the fraction of delivered oxygen that is consumed). Absolute CMRO_2_ was then determined by multiplying OEF by O_2_ delivery, computed as the product of resting CBF by arterial O_2_ content. In the absence of intersection in between the HC and HO curves, the voxel is said to have no solution and will later be excluded from any ROI or voxel average in *M*, OEF and CMRO_2_. Because of the low CNR of the ASL hyperoxic response, the GM-averaged value obtained from the post-T1-correction Δ%CBF_HO_ was used as an estimate of the whole-brain post-T1-correction Δ%CBF_HO._ Previous studies [3,13] also report using a whole-brain estimate of Δ%CBF_HO_, with the difference that, in the current paper, the value was computed for each participant. In the equation defining *M* (Gauthier and Hoge 2012, equation 7), the parameter α, which expresses the relationship between changes in blood flow and blood volume, was assumed to be 0.18 [20] while β, defining the non-linear dependence of changes in R_2_* on deoxygenated hemoglobin, was set to 1.5 [21]. The hemoglobin concentration [Hb] was assumed to be 15 g Hb/dl blood, although this can be readily measured using a venous blood draw. It was also assumed that oxygen consumption remained constant during periods of hypercapnia and hyperoxia (CMRO2_HC_ and CMRO2_HO_). The sensibility of QUO2 model-derived estimates to the assumed parameters was also evaluated, as detailed in the section ‘Accuracy of QUO2 model-derived estimates—sensitivity to systematic errors’.

### Tissue segmentation

Automated segmentation of gray matter (GM) from the anatomical scans was carried out using the FMRIB Software Library (FSL) [22]. Structural images were extracted from T1-weighted scans using the brain extraction tool (FSL’s BET). Then, a binary mask delineating the brain was created along with a probability mask of GM employing the automated segmentation tool (FSL’s FAST). Both were resampled to the resolution of the functional EPI scans.

#### Regions of Interest

**Fig 1:**
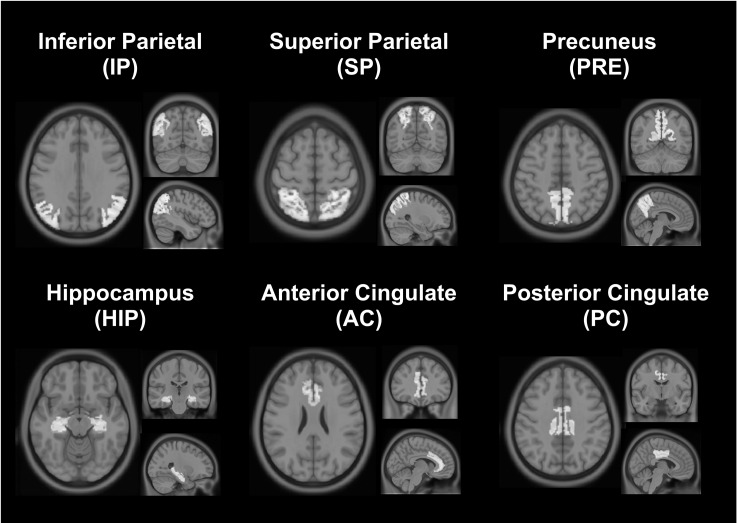
Regions of interest (ROIs). QUO2 parameters were evaluated in GM and in six ROIs selected from the OASIS-TRT-20 atlas.

In addition to the whole brain gray matter (GM), six ROIs in ICBM space were selected from OASIS-TRT-20 in three-dimensional mode [23]. The selected ROIs are located in parietal, occipital or temporal lobes, and are known to be implicated in conditions such as Alzheimer’s disease [24–29]. ROI’s, presented in Fig 1, include left and right: inferior parietal (IP), superior parietal (SP), precuneus (PRE), hippocampus (HIP), anterior (caudal and rostral) cingulate (AC) and posterior cingulate (PC). Each ROI was registered to the resolution of the functional EPI scans before being conjoined with the individual’s GM probability mask excluding voxels with a GM probability lower than 50% and non-parenchymal voxels identified previously. The resultant ROI probability masks were used to perform weighted averaging of the different metrics. Voxels where no solution was found for *M* and OEF were excluded when performing the ROI analysis of *M*, OEF and CMRO_2_.

#### Registration

Individual BOLD, CBF, *M*, OEF and CMRO_2_ maps were non-linearly normalized to the ICBM152 template using the CIVET software package [30] via the CBRAIN tool [31] with 12 degrees of freedom. Test A and B average maps of BOLD and CBF were computed as arithmetic means using in-house software. On their part, average maps of *M*, OEF and CMRO_2_ were obtained excluding from the average any voxels where no solution was found.

### Statistical Analysis

Within each measurement, i.e. CBF, BOLD (R2*), *M*, OEF and CMRO_2_, Test A and Test B were averaged and compared across ROIs, considering *P* < 0.05 level of significance, correction for multiple comparisons.

Statistical tests were performed on the data to ensure it satisfied the criteria for conducting a reproducibility analysis. For ETO_2_ and the other parameters listed above, the distribution of differences was tested for normality using the Shapiro-Wilk W-test, and the independence between the magnitude of differences and mean of measurements was verified using a rank correlation coefficient (Kendall’s τ). If the differences distribution appeared to deviate from a normal distribution, or if the magnitude of differences increased with the mean of measurements, the data were transformed on the log_10_ scale and the verification was repeated. In cases where the log_10_ scaled data satisfied the criteria, the reproducibility was assessed on these scaled values. Otherwise, assessment of reproducibility was based on the original values along with appropriate annotation [32–34]. Additionally, to determine whether there was an order effect between the two tests, we performed a two-tailed paired *t*-test on each set of ROI-averaged values, considering a *P* < 0.05 level of significance.

ROI-averaged reproducibility was evaluated using Matlab to compute metrics that give complementary information on the agreement between repeated measures and population variance:

1. dSD, the standard deviation of the differences between Test A and B measurements.
2. wsSD, the within-subject standard deviation, equals dSD/√2 considering two measurements.
3. wsCV, the within-subject (or intra-subject) coefficient of variation, as used in Floyd et al [33] and Chen et al [35]. wsCV = √ [mean of the (wsSD/subject mean)^2^]. wsCV provides an unbiased reproducibility measurement expressed as a percent of the mean with a low wsCV indicating a high reproducibility. When data were on the log_10_ scale, wsCV was approximated by antilog(wsSD)-1 [36].
4. CR, the coefficient of repeatability [37] = 1.96*√2*wsSD or 1.96*dSD. CR gives an estimate of the range of values one would obtain in a retest measurement. Thus, 95% of repeated measures for the sample will lie between the interval mean differences ± CR (⍺ = 0.05).
5. bsCV, the between-subject (or inter-subject) coefficient of variation as computed in Tjandra et al [38]. bsCV = SD_pooledData_ / mean_pooledData_ * 100.

### Accuracy of QUO2 model-derived estimates—sensitivity to systematic errors

The QUO2 derived estimates accuracy rely on assumed physiological parameters such as α, β, [Hb], as well as assumed normalized CMRO_2_ changes during HC and HO. As sources of systematic errors, the assumed values won’t affect the reproducibility/precision analysis outcomes, however they can lead to individual inaccuracy of *M*, OEF and CMRO_2_. Using the group-averaged GM experimental Test A data, the sensitivity of QUO2 estimates to assumed parameters was evaluated by independent variation in α, β, [Hb], CMRO2_HC_ and CMRO2_HO_. The values used in the previous analysis were: α = 0.18, β = 1.5, [Hb] = 15g Hb/dl blood, while isometabolism during hypercapnia and hyperoxia was considered (CMRO2_HC_ and CMRO2_HO_ = 1.0). Explored ranges of α and β were respectively 0.15 to 0.45 and 1.0 to 1.5, matching those in Chiarelli and al. [13]. The span of [Hb], i.e. from 11 to 17 g Hb/dl blood, was chosen as in Mark et al. [39] to take into account differences in gender and presence of anemia or polycythemia [40]. Evaluated ranges of change in CMRO_2_ during HC and HO were determined as in Merola et al [41]: i.e. a change of ± 1% in CMRO_2_ for 1 mmHg and 40 mmHg change in end-tidal CO_2_ and O_2_ respectively. We also evaluated the impact of a maximum of 10% decrease in blood flow during HO periods, as this parameter is often assumed (normalized CBF_HO_ from 0.90 to 1.0).

### Precision of QUO2 model-derived estimates—sensitivity to random errors

Within-subject precision of the QUO2 model-based *M*, OEF and CMRO_2_ estimates can be affected by a certain real physiological within-subject variability as well as random errors in the measurement of brain’s responses to hypercapnia and hyperoxia. To evaluate the effect, on OEF and *M* precision, of such errors in measurement, we performed an error propagation analysis of the QUO2 model employing Test A data. In addition to the analysis of errors in measured CBF during HC, also discussed in Gauthier and Hoge [5], we examined the impact of errors in measured R2* changes during both respiratory challenges. For each observed source of error, individual OEF and *M* values were computed based on ‘real’ GM and respiratory measures, with simulated error ranging from -33% to +33% added to the examined source. This simulated error can be translated into coefficient of variation of the observed input: the CV being the percent of variability with respect to the mean value, it is lower when an error is added to the measurement (CV of 20% for an error of +33%) rather than subtracted (CV of 28% for an error of -33%). Simulated group-average *M* and OEF were computed from the individual simulated values, while the latter were compared with the ‘real’ values to calculate the simulated CVs with respect to the added error.

## Results

One participant was excluded from the analysis because their CBF response to CO_2_ during Test A was found to be an outlier (value beyond twice the standard deviation). This participant reported a high level of anxiety during Test A due to a first MRI scan and hypercapnia experience.

### Gas manipulations

**Fig 2:**
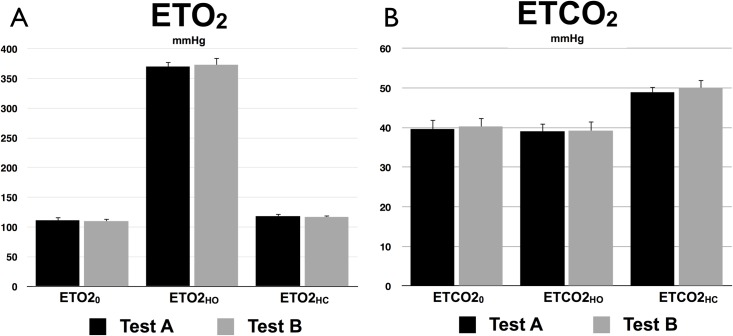
Gas manipulation. Test A and B end-tidal O_2_ and CO_2_ values at baseline (0), during hyperoxia (HO) and during hypercapnia (HC). Errors bars indicate standard deviation.

The average and standard deviation of ETO_2_ levels in Test A and B are shown in Fig 2A. Breathing medical air (~160 mmHg of O_2_) yielded average ETO_2_ levels of 111 ± 6 mmHg and 110 ± 4 for the two scan time points. During hyperoxia induction, when participants received a gas mixture with 380 mmHg of O_2_, ETO_2_ levels increased to 370 ± 9 mmHg during Test A and 374 ± 12 mmHg during Test B. During hypercapnia periods, participants demonstrated a slight increase in the minute volume ventilation, which increased ETO_2_ levels in comparison to air-breathing (118 ± 4 and 117 ± 4 mmHg) despite a slightly lower inhaled O_2_ concentration (~152 mmHg). Fig 2B shows the average and standard deviation of ETCO_2_ levels in Test A and B. ETCO_2_ levels at baseline and during hyperoxia were found to be similar, with 40 ± 2 mmHg and 39 ± 2 mmHg (in both tests) respectively. During HC, the ETCO_2_ levels increased to 49 ± 1 mmHg and 50 ± 2 mmHg in Test A and B respectively. Table 1 shows, for both ETO_2_ and ETCO_2_ measurements, *P*-values of Shapiro-Wilk W-test, Kendall’s rank coefficient and *t*-Student paired test, as well as wsCV and CR. All distributions of differences in tests were found to be normal (Shapiro-Wilk W-test, *P* > 0.05) and no dependence was observed between the differences in measurements and mean of measurements (Kendall’ τ, *P* > 0.2). Finally, no significant difference (*t*-Student paired test, *P* > 0.1) was found between Test A and Test B end-tidal levels, which were also reproducible (ETO_2_: CR<20 mmHG, wsCV<5%; ETCO_2_: CR<6 mmHG, wsCV<6%).

**Table 1:** End-tidal measurements.

	ETO2_0_	ETO2_HO_	ETO2_HC_	ETCO2_0_	ETCO2_HO_	ETCO2_HC_
**Test A mean ± SD**	111 ± 6	370 ± 9	118 ± 4	40 ± 2	39 ± 2	49 ± 1
**Test B mean ± SD**	110 ± 4	374 ± 12	117 ± 4	40 ± 2	39 ± 2	50 ± 2
**All mean ± SD**	111 ± 5	372 ± 10	118 ± 4	40 ± 2	39 ± 2	50 ± 2
**Shapiro-Wilk**	P = 0.38	P = 0.24	P = 0.25	P = 0.05	P = 0.3	P = 0.08
**Kendall’s τ**	P = 0.38	P = 0.24	P = 1.00	P = 0.38	P = 0.56	P = 0.38
**Paired Student’s t-test**	P = 0.73	P = 0.39	P = 0.51	P = 0.58	P = 0.83	P = 0.1
**CR**	13	19	10	5	3	4
**wsCV**	4.4%	1.9%	3.0%	5.0%	3.2%	2.6%

Group-averaged ± SD of end-tidal values are presented, followed by Shapiro-Wilk, Kendall’s τ and paired *t*-test *P* value. Coefficient of repeatability (CR) and within-subject coefficient of variation (wsCV) are also shown.

### Exclusion of non-parenchymal or artifact voxels

**Fig 3:**
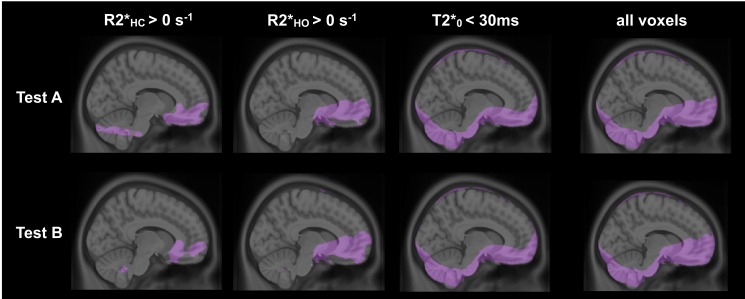
Exclusion of non-parenchymal or artifact voxels. For both tests, voxels considered as non-parenchymal or affected by susceptibility artifacts based on individual ΔR2*_HC_, ΔR2*_HO_ and T2*_0_ are shown overlapping a sagittal slice of the ICBM template. The latest column presents the overall excluded voxels in each test.

Fig 3 shows sagittal group-averaged maps of voxels excluded from the ROI analysis given that they were considered as non-parenchymal or dominated by susceptibility artifacts, based on individual ΔR2*_HC_, ΔR2*_HO_ and T2*_0_. Both tests presented similar patterns of excluded voxels, and, as expected, the latter were mainly situated in regions adjacent to the nasal cavity due to the paramagnetic effect of molecular O_2._ In Table 2, we present, for each ROI, the group average and standard deviation of the number of voxels with GM probability higher than 0.5, preceding and following the exclusion process. No significant difference was found between Test A and Test B number of pre- and post-exclusion voxels (all *P* > 0.23). In whole GM, 24 ± 4% were excluded from the analysis. The hippocampus and the anterior cingulate were the most affected by the procedure, with a percentage of exclusion of 31 ± 10% and 30 ± 9% respectively, whereas less than 2% of voxels were excluded in the other regions.

**Table 2:** Exclusion of non-parenchymal or artifact voxels.

	GM	IP	SP	PRE	HIP	AC	PC
**Number of voxels pre-exclusion**							
**Test A mean ± SD**	5676 ± 550	234 ± 33	197 ± 32	168 ± 33	79 ± 11	88 ± 21	59 ± 14
**Test B mean ± SD**	5715 ± 541	226 ± 21	204 ± 20	167 ± 23	78 ± 12	88 ± 15	53 ± 10
**Paired t-test**	P = 0.35	P = 0.26	P = 0.30	P = 0.90	P = 0.55	P = 0.95	P = 0.25
**Number of voxels post-exclusion**							
**Test A mean ± SD**	4331 ± 567	233 ± 33	193 ± 26	166 ± 32	54 ± 7	63 ± 23	59 ± 14
**Test B mean ± SD**	4316 ± 561	225 ± 22	200 ± 16	167 ± 23	53 ± 10	62 ± 16	53 ± 10
**Paired t-test**	P = 0.74	P = 0.23	P = 0.28	P = 0.92	P = 0.84	P = 0.93	P = 0.25
**Percent of excluded voxels (%)**							
	24 ± 4	0.58 ± 0.91	1.81 ± 2.37	0.60 ± 0.73	31 ± 10	30 ± 9	0.00 ± 0.00

Group-averaged ± SD of the number of voxels in each ROI are presented before and after the voxels exclusion procedure. Paired *t*-test *P* values between Test A and Test B number of voxels as well as percentage of voxels excluded are also shown.

### T1 shortening

Estimates of arterial blood T1 during HO were found to be 1.558 ± 0.004 sec for Test A and 1.556 ± 0.006 sec for Test B, with no significant difference between them (paired *t*-test, *P* = 0.39). Computation of GM-averaged post-T1-correction Δ%CBF_HO_ resulted in 0.4 ± 4.5% for Test A and -2.8 ± 3.3% for Test B. No significant difference (paired *t*-test, *P* = 0.25) was found between them and both were not significantly different from zero (*P* > 0.07).

### Detection rate of solutions in ROI

The percentage of voxels where a solution was found for *M* and OEF were computed for each individual and test. The results, presented in the Table 3, show no significant difference between Test A and Test B (*P* > 0.21) and all detection rates were above 87% besides in HIP where the detection rare were 78%. These percentages are based on the number of voxels after exclusion of non-parenchymal and artifact voxels found in the Table 2.

**Table 3:** Detection rate of solutions in ROI (%)

	GM	IP	SP	PRE	HIP	AC	PC
**Test A**	89 ± 5	95 ± 4	97 ± 3	93 ± 9	78 ± 11	87 ± 11	100 ± 1
**Test B**	88 ± 7	92 ± 10	95 ± 6	91 ± 11	78 ± 13	94 ± 5	99 ± 2
**Paired t-test**	P = 0.86	P = 0.53	P = 0.56	P = 0.73	P = 0.99	P = 0.21	P = 0.25

Group-averaged ± SD of the percentage of voxels where a solution was found for *M* and OEF are shown in each ROI. Paired *t*-test *P* values between Test A and Test B detection rate are also shown.

### Test and retest group-averaged metrics across ROIs

**Fig 4:**
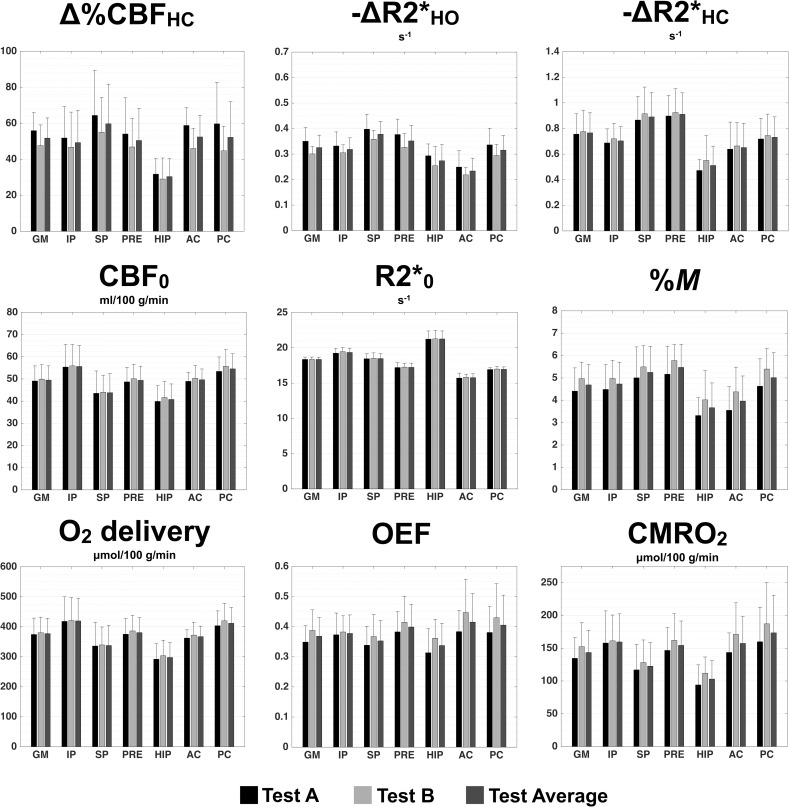
Test and retest group-averaged metrics across ROIs. ROI-averaged of Test A, Test B and all tests averaged are presented with standard deviation.

A summary of the dual-echo pCASL and QUO2 ROI-averaged metrics is presented in Fig 4. For each combination of metric and ROI, we present the group average of Test A, Test B and of both tests. Resting CBF, O_2_ delivery and resting R2* displayed the highest group-averaged test-retest reproducibility. Resting R2* presented the lowest population variance in both tests. Resting CBF and O_2_ delivery, due to their direct relation, showed a similar pattern of values across ROIs. Similarly for *M* and ΔR2*_HC_, given that QUO2 *M* values are very sensitive to change in R2* and CBF during HC while nearly insensitive to change during HO (as later demonstrated in the “Precision of group GM-averaged values–sensitivity to random errors” **section**). A larger decrease in R2* (higher BOLD increase) was observed during HC than during HO in every ROIs. Although every metrics, but OEF, showed a certain tendency of heterogeneous values across ROIs, all differences were not found statistically significant, as presented in the following section.

### Comparison of all tests averaged between ROIs

**Fig 5:**
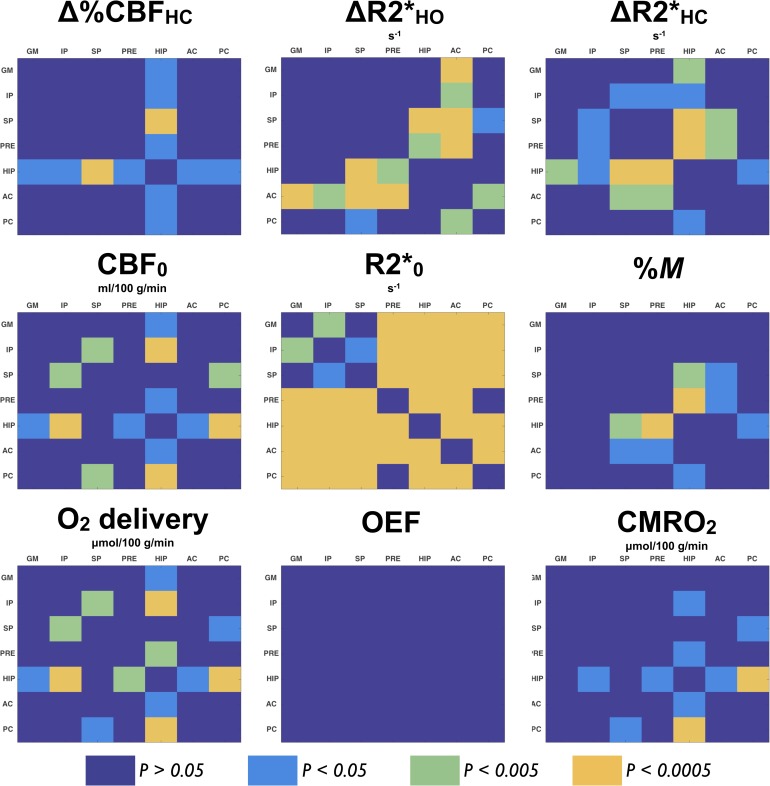
Comparison of all tests averaged between ROIs. For each metric, group average of all subjects and tests was compared across ROIs. Colors code for limits in *P* values after correction for multiple comparisons: dark blue indicates absence of significant difference (*P* > 0.05), while light blue (*P* < 0.05), green (*P* < 0.005) and orange (*P* < 0.0005) illustrate a significant difference between two ROIs (designed in the X and Y axis).

Data from all subjects and tests were averaged and compared across ROIs. Fig 5 shows where a significant difference (*P* < 0.05) was found between two ROIs, after correction for multiple comparisons. No statistical significant difference was found in fractional CBF change to HC across ROIs, except in the HYP, were the smallest change was found. R2* change to HO was found to be the smallest in AC among all ROIs except compared to HYP values, whereas the smallest R2* change to HC was found in HYP. Due to its low population variance within ROI, resting R2* presented values generally significantly different across ROIs. On the contrary, OEF were found to have consistent values across ROIs (*P* > 0.2).

### ROI reproducibility analysis

In Table 4, we report the reproducibility analysis of the dual-echo pCASL and QUO2 measurements in each ROI, more precisely, the *P*-value of Kendall’s τ, Shapiro-Wilk and paired *t*-test, the CR and wsCV. Shapiro-Wilk and Kendall’s τ test detected, for a certain combination of metric-ROI, if the distribution deviated from normality (condition *a* in Table 4) and if there was a dependency between the differences in measurements and the mean of measurements (condition *b* in Table 4) respectively. In all cases, a log_10_ transformation of the data resulted in the satisfaction of both conditions (reproducibility of transformed data is presented in boldface) except for Δ%CBF_HC_ in AC which distribution deviated from normality after the transformation (reproducibility of original data is presented in italic boldface). Paired *t*-tests detected no significant difference between Test A and Test B (*P* > 0.07), except for %*M* in AC (*P* < 0.001), OEF in HIP (*P* < 0.03) and CMRO_2_ in HIP (*P* < 0.05).

**Table 4:** Reproducibility of QUO2 measurements in different ROI.

	GM	IP	SP	PRE	HIP	AC	PC
**Average volume**							
**Mean ± SD, cm**^**3**^**>50% prob GM**	394 ± 49	20.9 ± 2.5	17.9 ± 1.9	15.2 ± 2.4	4.9 ± 0.8	5.7 ± 1.8	5.1 ± 1.1
**Δ%CBF**_**HC**_							
**Shapiro-Wilk**	P = 0.56	P = 0.19	P = 0.04^a^	P = 0.58	P = 0.78	P = 0.03^a^	P = 0.007^a^
**Kendall’s τ**	P = 0.77	P = 0.77	P = 0.14	P = 0.38	P = 0.24	P = 0.56	P = 0.24
**Paired Student’s t-test**	P = 0.19	P = 0.45	P = 0.43	P = 0.51	P = 0.68	P = 0.08	P = 0.23
**CR**	31.1	32.1	0.4	51.6	30.4	*38*.*8*	0.5
**wsCV**	22.4%	25.9%	35.6%	35.8%	40.3%	*26*.*5%*	46.9%
ΔR2*_HO_							
**Shapiro-Wilk**	P = 0.21	P = 0.51	P = 0.02^a^	P = 0.49	P = 0.65	P = 0.46	P = 0.93
**Kendall’s τ**	P = 0.24	P = 0.03^b^	P = 0.24	P = 1.00	P = 0.14	P = 0.07	P = 0.77
**Paired Student’s t-test**	P = 0.08	P = 0.41	P = 0.18	P = 0.17	P = 0.20	P = 0.21	P = 0.22
**CR**	0.15	0.21	0.17	0.18	0.15	0.12	0.17
**wsCV**	16.0%	18.9%	15.3%	18.9%	19.2%	17.4%	19.3%
ΔR2*_HC_							
**Shapiro-Wilk**	P = 0.57	P = 0.40	P = 0.47	P = 0.05	P = 0.21	P = 0.16	P = 0.02^a^
**Kendall’s τ**	P = 0.56	P = 1.00	P = 0.56	P = 0.77	P = 0.03^b^	P = 0.77	P = 1.00
**Paired Student’s t-test**	P = 0.49	P = 0.35	P = 0.13	P = 0.49	P = 0.12	P = 0.54	P = 0.37
**CR**	0.14	0.17	0.16	0.18	0.18	0.19	0.08
**wsCV**	6.70%	8.93%	7.43%	8.09%	16.10%	11.00%	6.93%
**CBF**_**0**_ **mL/100 g/min**							
**Shapiro-Wilk**	P = 0.90	P = 0.25	P = 0.93	P = 0.07	P = 0.80	P = 0.45	P = 0.39
**Kendall’s τ**	P = 1.00	P = 0.56	P = 0.56	P = 0.07	P = 1.00	P = 0.14	P = 0.56
**Paired Student’s t-test**	P = 0.48	P = 0.66	P = 0.77	P = 0.37	P = 0.13	P = 0.50	P = 0.31
**CR**	5.4	5.5	7.9	7.4	6.0	9.8	10.9
**wsCV**	3.88%	3.38%	6.50%	5.32%	5.23%	6.99%	7.45%
R2*_0_							
**Shapiro-Wilk**	P = 0.45	P = 0.10	P = 0.64	P = 0.19	P = 0.32	P = 0.46	P = 0.55
**Kendall’s τ**	P = 1.00	P = 0.77	P = 0.07	P = 0.38	P = 0.14	P = 1.00	P = 1.00
**Paired Student’s t-test**	P = 0.91	P = 0.07	P = 0.65	P = 0.42	P = 0.85	P = 0.36	P = 0.52
**CR**	0.17	0.65	0.55	0.33	0.94	0.56	0.45
**wsCV**	0.33%	1.24%	1.05%	0.70%	1.54%	1.30%	0.96%
**%*M***							
**Shapiro-Wilk**	P = 0.04^a^	P = 0.31	P = 0.05^a^	P = 0.91	P = 0.20	P = 0.08	P = 0.22
**Kendall’s τ**	P = 1.00	P = 0.24	P = 0.38	P = 0.77	P = 0.77	P = 0.77	P = 1.00
**Paired Student’s t-test**	P = 0.11	P = 0.19	P = 0.28	P = 0.23	P = 0.12	P = 0.0006^c^	P = 0.10
**CR**	0.19	1.84	0.22	2.50	2.32	1.72	2.42
**wsCV**	16.8%	16.6%	20.2%	18.1%	21.5%	16.7%	19.1%
**O2deliv μmol/100 g/min**							
**Shapiro-Wilk**	P = 0.86	P = 0.27	P = 0.76	P = 0.16	P = 0.29	P = 0.75	P = 0.42
**Kendall’s τ**	P = 1.00	P = 1.00	P = 0.56	P = 0.14	P = 1.00	P = 0.24	P = 0.56
**Paired Student’s t-test**	P = 0.49	P = 0.77	P = 0.79	P = 0.37	P = 0.21	P = 0.52	P = 0.34
**CR**	41	44	59	58	46	71	83
**wsCV**	3.87%	3.63%	6.29%	5.35%	5.36%	6.75%	7.43%
**OEF**							
**Shapiro-Wilk**	P = 0.12	P = 0.03^a^	P = 0.75	P = 0.19	P = 0.32	P = 0.09	P = 0.21
**Kendall’s τ**	P = 0.38	P = 0.24	P = 1.00	P = 0.24	P = 0.56	P = 1.00	P = 0.77
**Paired Student’s t-test**	P = 0.15	P = 0.76	P = 0.41	P = 0.35	P = 0.02^c^	P = 0.13	P = 0.37
**CR**	0.14	0.16	0.16	0.16	0.12	0.21	0.26
**wsCV**	13.6%	14.1%	16.5%	14.7%	14.7%	18.2%	23.0%
**CMRO**_**2**_ **μmol/100 g/min**							
**Shapiro-Wilk**	P = 0.47	P = 0.47	P = 0.58	P = 0.07	P = 0.55	P = 0.36	P = 0.26
**Kendall’s τ**	P = 0.56	P = 0.03^b^	P = 0.03^b^	P = 0.24	P = 0.77	P = 0.56	P = 1.00
**Paired Student’s t-test**	P = 0.15	P = 0.80	P = 0.51	P = 0.36	P = 0.05^c^	P = 0.10	P = 0.30
**CR**	62	0.16	0.25	80	50	88	128
**wsCV**	15.2%	14.0%	23.3%	17.9%	20.6%	18.9%	26.7%

P value of Shapiro-Wilk, Kendall’s τ, paired t-test, coefficient of repeatability (CR) and within-subject coefficient of variation (wsCV) are presented. When conditions described in footnote ^a^ or ^b^ were present, data were log_10_ transformed and the conditions were evaluated once again. If the conditions were then satisfied, the reproducibility metrics were computed on the transformed data (results presented in boldface). Otherwise, the reproducibility metrics were computed on the original data (results presented in italic boldface.

^a^ The distribution of difference in measurements deviated from normality.

^b^ A significant dependency between difference in measurements and mean of measurements was detected.

^c^ A significant difference between Test A and Test B was detected

**Fig 6:**
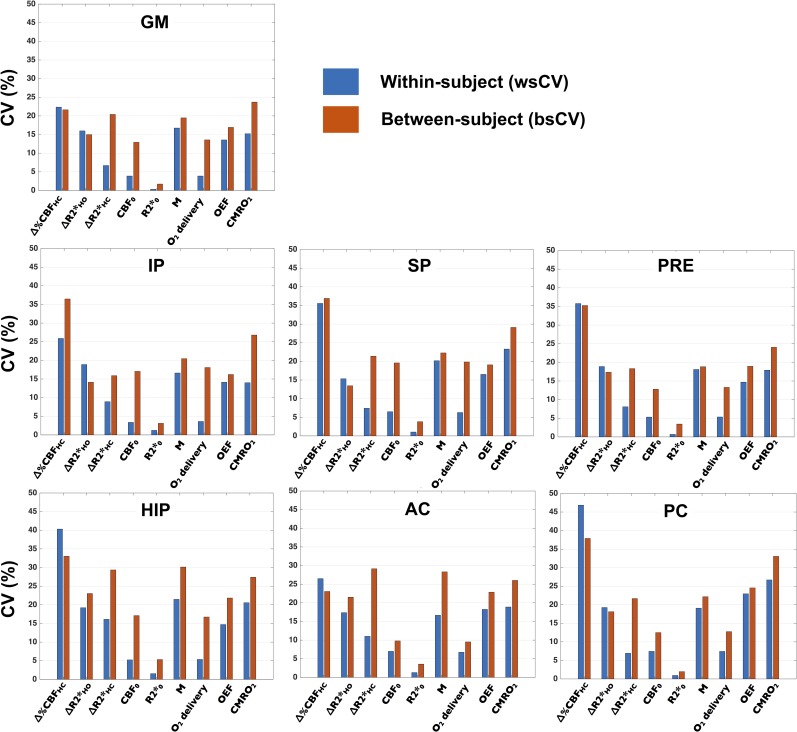
ROI analysis of within- and between-subject reproducibility. Within each ROI, are shown the within- and between-subject coefficient of variation (respectively wsCV (blue) and bsCV (red)) for each metric.

Fig 6 compares individual-subject reproducibility (wsCV) with the population variance (bsCV) of QUO2 measurements in each ROI. Among all ROIs, the measurement that showed lower inter- and intra-subject variability was resting R2* (wsCV < 2%; bsCV < 5.5%). CBF_0_ and O_2_ delivery come next, with a low individual-subject reproducibility (ROI-averaged wsCV = 5.5%) compared to the population variance (ROI-averaged bsCV = 14.6%), meaning the latter cannot be explained by poor reproducibility of the technique. It is also an indication of reproducible estimate of arterial O_2_ content. ΔR2_HC_ was also found to have a considerably higher intra-subject consistency than across subjects (ROI-averaged wsCV = 9.3%; ROI-averaged bsCV = 22.3%), as opposition to ΔR2_HO_ (ROI-averaged wsCV = 17.9%; ROI-averaged bsCV = 17.5%). The highest inter- and intra-subject variability was found in Δ%CBF_HC_ (wsCV_GM_ = 22.4%; wsCV_allROI_ = [22.4%,46.9%]; bsCV_GM_ = 21.6%; bsCV_allROI_ = [21.6%,37.9%]). As for the QUO2 estimated measurements *M*, OEF and CMRO_2_, their wsCV in GM were found to be lower than 17% and they ranged from 13% to 27% across all ROIs. Their variability across subjects were higher by a factor of 1.3 in average across ROIs. Values averaged over GM were found to have the lowest inter- and intra-subject variability among regional averages (mean wsCV of 11.0 ± 7.4%; mean bsCV of 16.15 ± 6.55%) whereas variability in smaller regions, i.e. HIP and PC, was generally found higher. A significant correlation between the variability of a certain metric and the volume of observed region was detected only for resting R2* measurement (Pearson correlation coefficient R_2_ = -0.76, *P* = 0.48).

**Fig 7:**
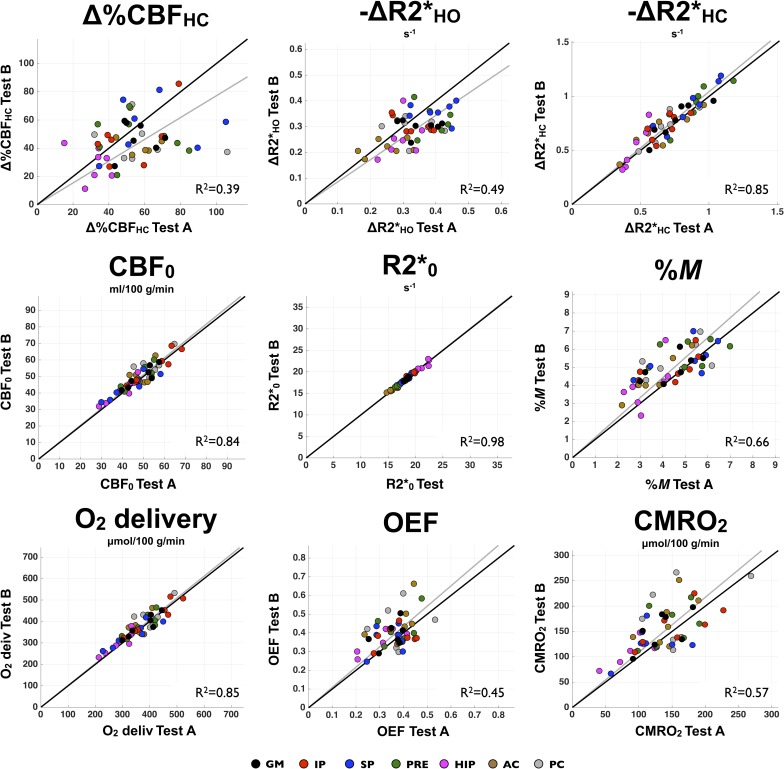
ROI analysis of individual reproducibility—Correlation plots. For each measurement, individual Test A against Test B ROI-averaged is plotted. Values close to the identity line (solid black line) indicate a good reproducibility. For each metric, a linear regression with zero intercept was performed on the data. The resulted coefficient of detection (R^2^) and the fit to the data (gray line) are presented.

Test-retest correlation plots for each metric across the different ROIs can be found in Fig 7. For each individual, Test A against Test B measurements is shown, with colors coding for the different ROIs. For each metric, a linear regression model with zero intercept was applied on the data. The computed coefficient of determination (R^2^) is displayed in each graph. In each measurement, the regression line that fit the data is shown in solid gray. Values lying in the vicinity of the identity line (solid black line) indicate a good reproducibility of the measurements. Among all quantities, resting R2*, resting CBF, O_2_ delivery and ΔR2*_HC_ present the best reproducibility, whereas Δ%CBF_HC_, ΔR2*_HO_, %*M*, OEF and CMRO_2_ show a larger scattering of the data around the identity line.

**Fig 8:**
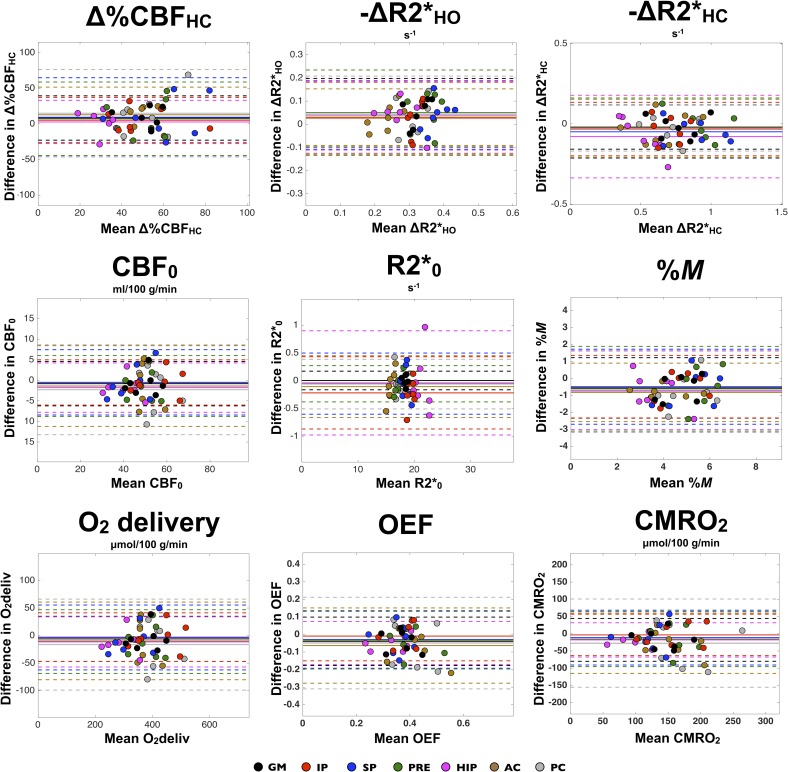
ROI analysis of individual reproducibility–Bland-Altman graphs. For each measurement, the difference in sessions against the mean is displayed. Within each ROI, a solid line represents the mean difference between measurements. The dashed lines represent the ROI-averaged limits of agreement (mean ± 1.96 * dSD i.e. mean ± coefficient of repeatability (CR)) indicating that 95% of repeated measures will fall in between them.

An additional illustration of reproducibility is offered by Bland-Altman plots for each measurement, as shown in Fig 8. The individual mean of measurements against the difference in measurements is plotted, with colors coding for the different ROIs. For each ROI, a solid line represents mean of difference, whereas dashed lines depict the confidence intervals (i.e. mean difference ± CR). We can observe a trend toward higher variability in smaller regions (HIP, AC, and PC) compared to larger regions.

### Parametric maps

**Fig 9:**
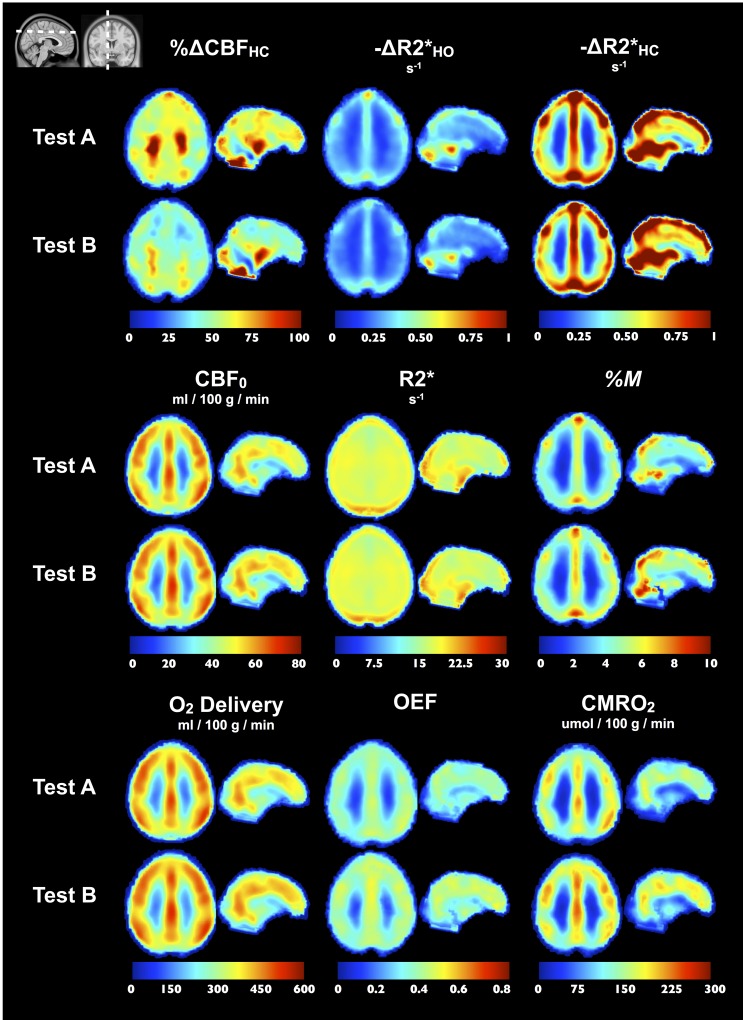
Group maps. For each metric, Test A and B group maps are shown in one axial and one sagittal slice. Maps were non-linearly registered to ICBM152 before being averaged. *M*, OEF and CMRO_2_ maps were averaged using an approach where non-solution voxels were excluded from the average.

**Fig 10:**
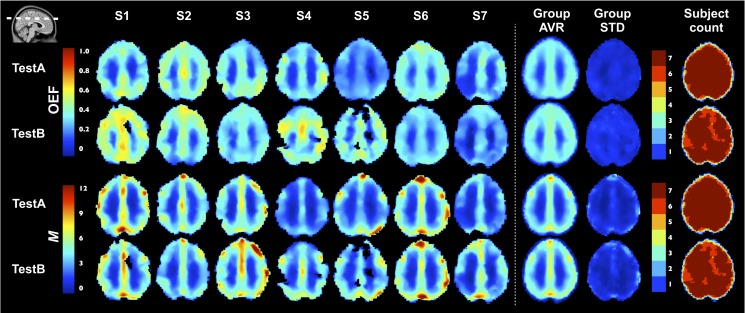
Individual OEF and M maps. Test A and B individual OEF and *M* maps are shown in one axial slice of ICBM152 template. Followed maps are the group average, the standard deviation, and the subject count maps. The latter maps indicate at each voxel the number of subjects where a solution was found for OEF and *M*, meaning they were part of the group average.

In Fig 9, we show group-average Test A and B maps of the different measurements. One axial and one sagittal slice of the ICBM152 template are shown. The group-average maps generally demonstrate good qualitative agreement between test and retest, with the main exception being Δ%CBF_HC_. Fig 10 shows OEF and *M* maps in individual subjects, for Tests A and B, as well as group average and standard deviation maps. One axial slice of the ICBM152 template is shown. The subject count maps indicate, at each voxel, the number of subject included in the group average, given that voxels with no solution found for OEF and *M* were excluded from the average. Individual OEF and *M* maps generated using the optimized QUO2 present little random fluctuation in values and very few voxels with no solution. Group average maps for both parameters were qualitatively very similar in appearance, although differences between Tests A and B are noted in individual subjects.

### Accuracy of group GM-averaged values—sensitivity to systematic errors

**Fig 11:**
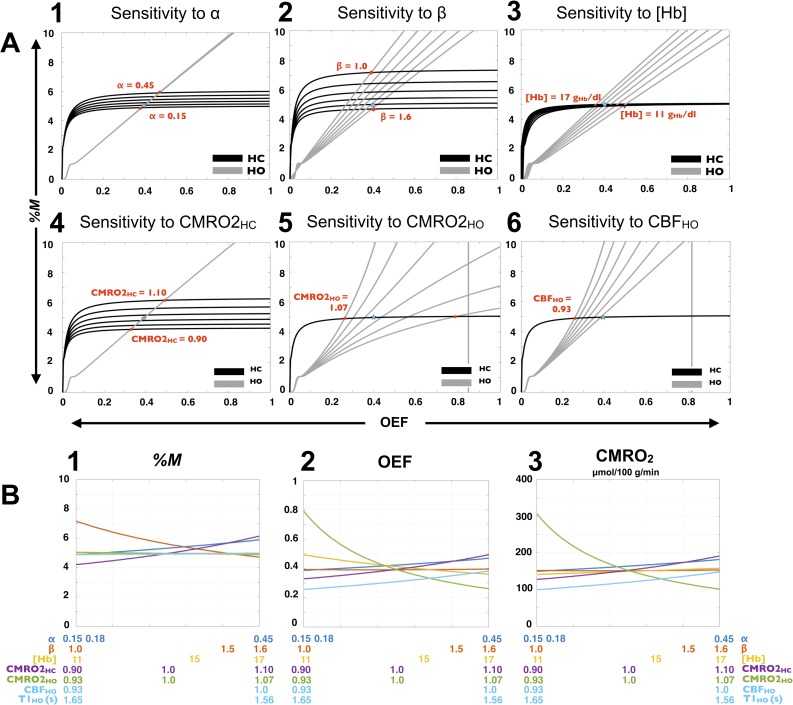
Accuracy of group GM-averaged values—sensitivity to systematic errors. Observed effects, on *M*, OEF and CMRO_2_ estimates, of the assumed QUO2 parameters, are summarized. Estimates were based on group-averaged Test A de-PCASL measurements in GM and ETO_2_ (‘true’ values), while the assumed parameters α, β, [Hb], normalized change in CMRO_2_ due to HC (CMRO2_HC_) and HO (CMRO2_HO_), as well as normalized change in CBF during HO (CBF_HO_) were varied independently. The HC and HO curves resulting from the use of six different values of α, β, [Hb], CMRO2_HC_, CMRO2_HO_ and CBF_HO_ are presented (A1 to A6 respectively). Each red dot represents the HC and HO curves intersection (hence one *M* and OEF solution) when either one of the extremity in the observed range is in use. The remaining *M* and OEF solutions lie on a line connecting both red dots and passing by the subsequent intersections. *M*, OEF and CMRO_2_ estimates are presented in function of each varied parameter (B1, B2 and B3 respectively, ranges mentioned below each plot). Below the CBF_HO_ range is shown the corresponding range of blood T1 during HO. The original values employed in this study were α = 0.18, β = 1.5, [Hb] = 15 g Hb/dl blood, CMRO2_HC_ = 1 (isometabolic hypercapnia), CMRO2_HO_ = 1 (isometabolic hyperoxia), while our post-T1-correction group-averaged normalized CBF_HO_ in Test A was estimated to be 1 (i.e. no CBF change during HO). Resulted ‘true’ group-averaged Test A *M*, OEF and CMRO_2_ estimates in GM were 4.96%, 0.39 and 152 μmol/100 g/min respectively (shown by the blue stars).

Sensitivity of the model to systematic errors resulting from the assumed parameters is summarized in Fig 11. Based on our Test A group-averaged changes in ET during each respiratory manipulation, our evaluated ranges of percent change in metabolism were ±10% and ±7% in HC and HO respectively. Effects of the assumed α, β, [Hb], CMRO2_HC_, CMRO2_HO_ and CBF_HO_ on the HC and HO curves are presented (Fig 11A1 to 11A6 respectively), followed by resultant estimates of *M*, OEF and CMRO_2_ (Fig 11B1, 11B2 and 11B3 respectively). The β and CMRO2_HC_ assumed values are the principal sources of variation in *M*, while variations in [Hb], CMRO2_HO_ and CBF_HO_ yield almost no change to the estimate. Fig 11A5 and 11A6 show that, by not affecting the HC curve, variations in metabolism and blood flow during HO shift the HO curve on the nearly horizontal section of the HC curve, resulting in almost no alteration in *M* solution. The assumption of change in CMRO_2_ during HO is the principal sources of variation in OEF, especially if a decrease in metabolism is considered (0.93 < CMRO2_HO_ < 1.0). Furthermore, OEF is similarly sensible to variation in [Hb], CMRO2_HC_ and CBF_HO,_ while presenting almost no change as a variation in β. Both *M* and OEF are similarly influenced by a variation in α. The used value of 0.18 for α results in an *M* and OEF of 5.0% and 0.39 respectively, while if α is changed to the commonly adopted values of 0.38, the calculated *M* and OEF values become 5.6% and 0.44. Estimate of metabolism being the product of resting CBF by OEF and arterial O_2_ content, a certain percent increase/decrease in OEF leads to the same percent increase/decrease in CMRO_2_ with the exception where [Hb] is varied, given that it leads to a change in the arterial O_2_ content in the opposite direction as in OEF.

### Precision of group GM-averaged values—sensitivity to random errors

**Fig 12:**
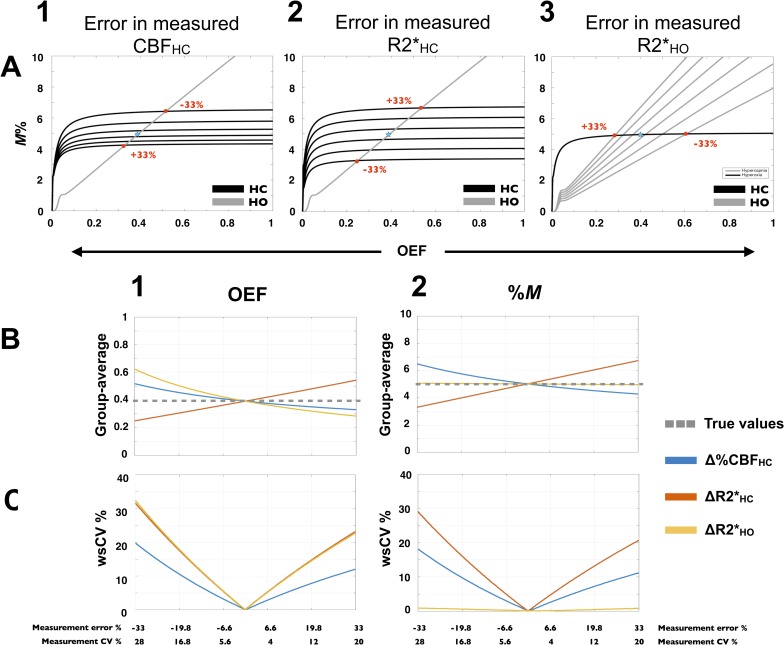
Precision of group GM-averaged values—sensitivity to random errors. Observed effects, on *M* and OEF estimates, of error in BOLD and CBF measurements, are summarized. Estimates were based on group-averaged Test A de-PCASL measurements in GM and ETO_2_ (‘true’ values), while a simulated error was added to each observed input independently. Evaluated error ranged from -33% to 33%, which is equivalent to a maximum measurement CV of 28% (when underestimated) and 20% (when overestimated). The HC and HO curves resulting from six different errors in CBF_HC_, BOLD_HC_ and BOLD_HO_ are shown (A1, A2 and A3 respectively). Each red dot represents the HC and HO curves intersection (hence one *M* and OEF solution) when either one of the extremity in the observed range is in use. The remaining *M* and OEF solutions lie on a line connecting both red dots and passing by the subsequent intersections. OEF and *M* estimates (B1 and B2 respectively) in addition to CV between simulated and ‘true’ OEF/*M* values (C1 and C2) were computed for each source of errors (colored lines). Without addition of error in measurements, ‘true’ group-averaged Test A *M* and OEF estimates were 4.96% and 0.39 respectively (shown by the blue stars and the perforated grey lines).

Sensitivity of *M* and OEF to random errors in BOLD and CBF measurements is summarized in Fig 12. Given that neither resting CBF nor arterial O_2_ content are varied here, the relative impacts of errors in BOLD and CBF responses measurements on CMRO_2_ and OEF are equivalent. Therefore, estimates and variation of CMRO_2_ are not shown. Effect of errors in CBF_HC_, R2*_HC_ and R2*_HO_ on HC and HO curves are presented in Fig 12A1, 12A2 and 12A3 respectively. Errors in CBF_HC_ or R2*_HC_ affect both *M* and OEF estimates, whereas errors in R2*_HO_, by shifting the HO curve on the nearly horizontal section of the HC curve, result in almost no alteration in *M* solution. OEF and *M* estimates as a function of percent errors or variability of measurement are shown in Fig 12B1 and 12B2 respectively. Fig 12B1 exhibits a nearly linear increase in OEF errors as each of the input’s error increases. Fig 12B2 presents the same observation for *M* estimates, with the exception mentioned above where errors in R2*_HO_ have almost no noticeable effect. The direction of the effect on OEF and *M* estimates depend of the source of error: an underestimated R2*_HC_ will lead to an underestimated OEF or *M*, whereas if overestimated, OEF and *M* will also be overestimated. On the other hand, CBF_HC_ have the opposite effect on OEF and *M* as well as R2*_HO_ on OEF. Fig 12C1 and 12C2 show resulted variability of OEF and *M* estimates respectively. We observe that for the same percent error, CBF_HC_ will induce a lower variability in OEF than R2*, and a lower variability in *M* than R2*_HC_. However, it is known that one of the limiting factors of such calibrated MRI methods is the low signal-to-noise ratio of ASL measurements, which is more susceptible to induce higher errors than in BOLD measurements. Fig 12C bring our attention to the fact that the test-retest variability found in our experimental CBF_HC_ (wsCV = 22%) and R2*_HO_ (wsCV = 16%) data, likely contributed to a minimum of 10% variability in our *M* and OEF estimates.

## Discussion

We have characterized the inter- and intra-subject variability of the estimated *M*, OEF and absolute CMRO_2_ derived from the optimized QUO2 approach, which is a crucial step in the development and clinical application of this MRI technique for the quantitative measurement of oxygen delivery and consumption in the human brain. A visual inspection of the individual maps of *M* and OEF revealed a larger stability of values and fewer regions with no solution than the individual maps presented in the initial proof-of-concept of the QUO2 model [6].

We obtained physiologically plausible tests-averaged *M*, OEF and CMRO_2_ estimates in GM that were compared to those in literature summarized in Table 5. Variability among different studies in estimated values can arise from: the imaging technique employed, the type of breathing manipulations, the values of the assumed parameters, and the strategy employed to define the grey-matter region. Our *M* average of 4.69 ± 0.91% falls in the low end of reported values, although this may be mainly due to a more aggressive exclusion of large venous voxels. Our OEF and CMRO_2_ of 0.37 ± 0.06 and 144 ± 34 μmol/100 g/min respectively fall within the reported range of values.

**Table 5:** Literature values of *M*, OEF and resting-state CMRO_2_.

Study	M	Study	OEF	CMRO2
	**(%)**			**μmol/100 g/min**
**Bulte et al. (2009) [42]**	5.3	**Xu et al. (2009) [50]**		158**
**Ances et al. (2008) [43]**	5.7	**Bolar et al. (2011) [51]**	0.26 ± 0.02	125 ± 15
**Gauthier et al. (2012) [6]**	6.0	**Gauthier et al. (2012) [6]**	0.35 ± 0.04	145 ± 30
**Ances et al. (2009) [44]**	6.5	**Lu and Ge (2008) [52]**	0.35 ± 0.06	
**Mark et al. (2011) [39]**	6.5–9.7	**Jain et al. (2010) [53]**	0.35 ± 0.01	123 ± 4
**Chen and Parrish (2009) [45]**	6.7	**Van Zijl et al. (1998) [54]**	0.36	137
**Wise et al. (2013) [4]**	6.9–9.2	**Wise et al (2013) [4]**	0.42–0.50	184±45–222±82
**Chiarelli et al. (2007) [13]**	7.0	**Coles et al. (2006) [55]**	0.38 ± 0.68	130 ± 24**
**Ances et al. (2009) [44]**	7.3	**Leenders et al (1990) [56]**	0.38	155*
**Bulte et al. (2012) [2]**	8.5	**Bulte et al. (2012) [2]**	0.38 ± 0.14	155 ± 39
**Gauthier et al. (2011) [46]**	9.5	**Bremmer et al. (2010) [57]**	0.43 ± 0.63	120 ± 8
**Lin et al. (2008) [47]**	10.5	**Ibaraki et al (2010) [58]**	0.43	137*
**Leontiev and Buxton (2007) [48]**	11.1	**Ito et al. (2004) [59]**	0.44 ± 0.06	129 ± 20
**Perthen et al. (2008) [49]**	11.6			

The majority of M values were taken from Gauthier et al [46]. All are values at 3 T, adjusted to TE = 30ms.

Pet studies are identified with a light grey background.

* Extrapolated value to match our mean age of 30 years old.

** Corrected value for the ratio of CMRO_2_ and mass between GM and WM [4].

### Regional inter- and intra-subject reproducibility

Our individual-subject reproducibility in every parameters was found to be lower than our population variance, as also reported in previous study [38,47,60], except for our CBF response to HC and BOLD response to HO. Inter- and intra-subject variability of QUO2 measurements was found to be the lowest when averaged throughout all GM, with general trends toward higher CVs when averaged over smaller regions, a finding that is to be expected based on statistical considerations and consistent with previous studies [60,61]. The most intra-subject reproducible measurements was CBF_0_, along with O_2_ delivery and R2*_0_. Our regional CBF_0_ wsCV were comparable to that reported by Wang et al [61].

To our knowledge, reproducibility of OEF and absolute CMRO_2_ estimated from combined HC and HO calibrated BOLD technique has not been characterized in studies so far published. On the other hand, variability of *M* calibration parameter and relative change in CMRO_2_ were studied. In a small area of the visual cortex, Leontiev and Buxton [48] evaluated the intra-subject variability in *M*, CBF and BOLD responses to HC employing a different CV computation. Adjusting our CV computation to theirs, we obtain a similar wsCV for *M* of 13% in GM (compared to 12.3%) and a lower variability in BOLD_HC_ and CBF_HC_ with 7% vs. 26.1 and 20% vs. 40.6% respectively. They reported the presence of an important leak in the CO_2_ delivery during day 2 for two of their participants, thus increasing their intra-subject variability in BOLD and CBF responses to HC. In another study at 7 Tesla, using a simultaneous hypercapnia-hyperoxia (carbogen) gas-breathing challenge, Krieger et al [60] detected more intra-subject variability in their *M* value and BOLD changes to carbogen than what we found (wsCV≃34% vs. wsCV<17%) while they reported less variable CBF changes to carbogen than our CBF changes during HC (wsCV≃9% vs. wsCV = 22%). The difference among variability in *M*, CBF and BOLD changes to gas may arise from the different choice of MRI acquisition and gas challenge.

Studies employing dynamic ^15^O PET as a direct measure of OEF and CMRO_2_ have reported lower intra-subject variability in OEF and CMRO_2_. Coles et al [55] reported a whole brain test-retest coefficient of variation of 4.6% and 3.7% for OEF and CMRO_2_ respectively, while Bremmer et al [57] obtained a variability of 8.8%, 9.3% and 5.3% for CBF_0_, OEF and CMRO_2_ in GM respectively. In our study, resting CBF in GM varied less (wsCV = 3.9%) while OEF and CMRO_2_ had a higher degree of variability (13.6% and 15.2% respectively). Higher intra-subject variability in QUO2 estimates than PET estimates may arise partly from a larger noise accumulation in QUO2 techniques as an indirect measure of OEF and CMRO_2_.

### Accuracy of group GM-averaged values—sensitivity to systematic errors

The QUO2 sensibility to assumed physiological parameters α, β, [Hb], CMRO2_HC,_ CMRO2_HO_ and CBF_HO_ was examined. Recently, Wise et al. employed a Bayesian estimation framework in order to estimate α and β [4]. Merola et al [41] presented a simplified calibration model that substitute the standard α and β parameters with a single one and yielded improved estimates of OEF. Accuracy of estimates is likely to benefit from those calibration techniques where α and β are automatically estimated. Individual OEF estimates can also benefit from a simple blood test to determine the hemoglobin concentration. Isometabolism during hypercapnia and hyperoxia remains of a debate to date. Some studies suggest that CMRO_2_ does not change with HC [62–64] while others report an increase [65], or a decrease [66,67]. Similarly, heterogeneous results are found with regard to metabolism alteration during hyperoxia [68]. Without a clear consensus on the matter, we opted to preserve the assumption of no change in CMRO_2_ during both respiratory manipulations.

We have reported a certain variation in OEF (and CMRO_2_) as a function of reduction in blood flow caused by the hyperoxia manipulation. It is common to assume a fixed T1-corrected CBF_HO_ for every subjects, for example Bulte et al. assumed a blood flow decrease of 4% during periods of hyperoxia at 50% O_2_ [2,69], or, as in the initial proof-of-concept of the QUO2 model, to compute the group-averaged measured CBF_HO_ after T1-correction and use it for every subject. In the present study, we obtained physiologically plausible arterial blood T1 values (1.56 sec at 60% O_2_, consistent with 1.49 sec at 95% O_2_ [70]) and post-T1-correction CBF_HO_ in GM. The latter was used as an individual basis, hence capturing any intra-subject variation between test and re-test blood flow decrease during HO. When correcting for T1 shortening during HO, rat blood was used as a surrogate for human blood which is justified since they both possess similar blood constitution, are likely to have very similar longitudinal relaxivity to molecular oxygen relationship and to experience similar physiological responses to hyperoxia.

### Precision of group GM-averaged values—sensitivity to random errors

The error propagation analysis highlighted how the precision in QUO2 estimates was affected by random errors in the measurements of CBF and BOLD responses to the respiratory challenges. Especially, considering the same range of error, BOLD had a larger impact on QUO2 precision than CBF. However in practice, CBF response to HC is more challenging and carries a bigger uncertainty due to the low contrast-to-noise ratio. The range of error observed (-33% to +33%) is likely to approach the order of error in measured CBF_HC_ while a lower uncertainty is expected in BOLD measurement, yielding smaller effects on QUO2 precision errors. It is therefore believed that the principal limitation in the QUO2 *M*, OEF and CMRO_2_ intra-subject reproducibility remains the error in CBF responses. Although the exact contribution of the measurements errors compared to the intrinsic day-to-day physiological variability could be further studied with a within-session test-retest reproducibility design, the present study offers clinically relevant assessment of variability in QUO2 measurements for follow-up studies.

### QUO2 qualitative individual and group maps reproducibility

As expected, the reproducibility of QUO2 parameter estimates was generally improved with more extensive averaging of voxels and subjects. While group average maps demonstrated qualitatively good reproducibility, it was found that physiological and measurement noise still limits reproducibility of voxel values at the individual subject level.

### Potential QUO2 accuracy and precision improvement

There are other potential avenues to improve the accuracy and precision of functional ASL. One strategy may include the use of a higher magnetic field, which would increase the SNR by increasing spin polarization and the T1 relaxation time of arterial blood. The gain in SNR might be partly lost due to an increase in physiological noise, however, this effect could be diminished by applying a denoising technique like RETROICOR [71,72]. Improved control for magnetization transfer effects is another mechanism for improving accuracy [73]. Additional approaches may also enhance accuracy and reproducibility of QUO2 measurements by correcting for region-specific variations in tag arrival times [74–76], for drops in label efficiency [77,78], for partial volume [79] and hemodynamic response delays [80]. Benefits may also be offered by improved imaging readout methods such as the use of a 3D imaging readout [81], multiband excitation [82] and background suppression [83–85].

Additional factors that can influence accuracy and precision of measurements in such calibrated approaches are the inhaled concentrations of oxygen and carbon dioxide. A higher O_2_ and CO_2_ concentration would yield BOLD changes closer to the *M* value to be extrapolated, reducing measurement errors. Furthermore, higher CO_2_ concentration would have the advantage of increasing the contrast-to-noise ratio due to higher CBF changes. However, administration of high levels of O_2_ and CO_2_ introduce potential problems: higher O_2_ concentration can complicate the quantification of CBF due to blood T1 reduction and susceptibility artifacts in areas close to sinuses and airways, while higher levels of CO_2_ can lead to anxiety and potentially alter brain physiology in ways other than the intended vasodilatory effect [86,87]. Future studies should assess the effect of oxygen and carbon dioxide concentration in reproducibility of quantitative calibrated methods such as QUO2. An investigation on the effect of O_2_ concentration in QUO2 estimates is presently in process, comparing 60% vs. 100% O_2_ during hyperoxia periods (manuscript in preparation).

The imaging parameters of the de-pCASL used in the present study were adjusted to optimize the detection rate of CBF responses in GM while acquiring whole-brain image with a minimal gap between slices. In order to allow the labeled blood bolus to arrive in the tissue of the imaged region, a post labeling delay (PLD) is employed. In our 2D acquisition, the first and last slices are acquired after a delay of 900 msec and 1986 msec respectively, resulting in an brain-averaged PLD of 1443 msec which is shorter than that recommended in the ASL white paper [16]. The latter recommends a 3D readout PLD of 1800 msec for healthy subjects of age below 70 years old. MacIntosh et al [74] evaluated the regional arterial time transit (ATT) in a cohort of healthy participants with range of age equivalent to that of our group. Among the temporal, parietal, frontal and occipital lobes, only the latter was reported to have a mean ATT (935 ± 0.108ms) slightly higher than our nominal PLD (900 msec), but lower than the acquisition time of our second slice (953 msec). Although our choice of PLD might not be optimal in lower occipital region of certain healthy participants, we believe that in the large majority of cases, the acquired ASL signal was accurately reflecting CBF and that an increase in our PLD would have resulted in a loss in SNR, especially in hypercapnic situation where the ATT is known to diminish.

Small cohort sizes like 8 have been common in recent years, particularly for complex fMRI protocols with greater physiological specificity than the classic BOLD contrast. Although our conclusion are limited by the relatively small sample size, we felt that this regional analysis of inter- and intra-subject variability of the QUO2 estimates in such a cohort remains of interest.

In conclusion, the variability of the optimized QUO2 estimates across subjects and the intra-subject reproducibility of estimates in different brain regions were characterized while the impact of errors on the accuracy and precision of such estimates was determined. These results will help guide power analyses for research applications as well as future developments aimed at further improving the reproducibility of the QUO2 method.
